# Burden of caring for children living with human immunodeficiency virus in a semi-rural South African community

**DOI:** 10.4102/safp.v62i1.5110

**Published:** 2020-06-25

**Authors:** Stacy Maddocks, Verusia Chetty

**Affiliations:** 1 Discipline of Physiotherapy, School of Health Sciences, University of KwaZulu-Natal, Durban, South Africa

**Keywords:** HIV, children, disability, burden of care, South Africa

## Abstract

**Background:**

Caregiver burden influences the well-being of children living with human immunodeficiency virus (HIV) who may experience disabilities as a result of the virus, comorbidities and treatment. Overall health, psychological well-being, finances, social life and the relationship with the child being cared for influence the burden of care. This study aimed to investigate the burden of care on caregivers of children living with HIV who may be experiencing disabilities.

**Methods:**

An analytical cross-sectional survey using the Zarit Burden questionnaire was conducted with caregivers of children living with HIV who were accessing care from a semi-rural healthcare setting between May and August 2019. A socio-demographic survey supplemented the Zarit Burden instrument. Descriptive statistics were used to determine burden of care and associations between the demographic profile variables of caregivers and the burden of care, with significance set as *p* < 0.05.

**Results:**

Thirty-eight caregivers completed the survey. Although 44.7% reported no burden of care, 36.8% reported mild-to-moderate burden and 18.4% moderate-to-severe burden of caring for children living with HIV. The only significant association was between caregiver health status and burden of care (*p* = 0.034).

**Conclusion:**

Although the burden of care in caregivers ranged between mild to severe and was directly associated with the caregiver’s health status, the findings of this study highlight a need to assess caregiver burden in all caregivers of children living with HIV so that appropriate referral to professionals for counselling and support can be initiated. Because caregiver burden affects the care offered to children, professionals need to integrate their well-being into healthcare.

## Introduction

Children living with human immunodeficiency virus (CLHIV), and experiencing disabilities, require appropriate and committed adult care to foster their development and well-being as they confront the multi-faceted challenges associated with living with the virus.^1,2^ Children living with HIV, and experiencing disability, face challenges related to inclusive education, making friends as well as access to healthcare.^3^ Caring for a child living with HIV and experiencing disability requires a substantial amount of financial, physical and emotional investment from the primary caregiver, including the provision of nutritious food, timeous administration of antiretroviral therapy (ART), adult accompaniment to monthly scheduled healthcare appointments and an attentive, nurturing family environment.^4,5,6,7^

The burden of caring for children with other chronic diseases or disability has been widely documented and can be described through the physical, emotional, social and financial challenges experienced by family members in their role as caregivers.^8^ Caregiver burden poses a significant risk to the overall well-being of not only the carers but also the children under their care.^9,10^ In South Africa (SA), family caregiving is mostly informal and unpaid.^4^ The chronicity of HIV, combined with its transgenerational probability, complicates traditional family caregiving roles, as opposed to other chronic childhood conditions like cerebral palsy or even cancer.^7^ Parents of CLHIV in SA are typically challenged by their own illness, financial hardship because of unemployment and reduced family support owing to the stigma and cultural beliefs around the virus.^4^ In the event of significant parental illness or death, grandparents or extended family members are expected to assume the responsibility of caring for CLHIV, usually under severe resource restraints.^7,11^ Consequently, caregivers of CLHIV are at risk of increased burden, which predisposes the children to reduced levels of care based on the aptitude, competencies and resources of the caregiver.^5^

Studies investigating the experiences and needs of caregivers of CLHIV recommend that caregivers be routinely screened for burden to implement interventions that support the well-being of the carer.^9,10,12^ This study is part of a larger study that aims to assess the feasibility (acceptability, practicality, preliminary efficacy) of an integrated model of rehabilitation and paediatric HIV care to improve the diagnosis of, and interventions for, disability in CLHIV between the ages of 5 and 10 years.^13^ In this article, researchers sought to determine the level of burden among caregivers of CLHIV, possibly experiencing disability, and who were accessing care at a semi-rural healthcare facility. The authors acknowledge that improving the care offered to the children begins with attending to the needs of the caregivers.

## Methods

This study used a quantitative cross-sectional design using the Zarit Burden Interview (ZBI) to measure the perceived care burden among caregivers of CLHIV between the ages of 5 and 10 years, accessing care at a single-site, district-level hospital in a semi-rural setting in KwaZulu-Natal. A disability prevalence study at the study setting^14^ revealed that a large number of children aged 5–10 years living with HIV were experiencing disabilities. Using non-probability sampling, caregivers of 58 children between the ages of 5 and 10 years, accessing care at this study site, were recruited during their routine monthly HIV clinic visits to the hospital. Participants had to be the primary caregiver responsible for the welfare of the child living with HIV, older than 18 years, and willing to be part of this study.

### Data collection

Informed consent was obtained from all caregivers and they were guaranteed that their participation would be strictly voluntary, with no incentives offered or withdrawal of care threatened. This study was conducted over a 4-month period (May–August 2019). Thirty-eight caregivers who fulfilled the inclusion criteria consented to participate.

The questionnaire, although developed to measure burden in caregivers of people living with Alzheimer’s disease, has been utilised to measure caregiver burden in other South African HIV studies.^10,15^ The ZBI was subjected to back-and-forth translations into isiZulu and English by a language specialist at the University of KwaZulu-Natal. Trained interviewers conducted individual face-to-face interviews with participants in English or IsiZulu, according to the patient’s preference. The tool measured burden of care according to five domains, namely, health, psychological well-being, finances, social life and relationship with the child, on a 5-point Likert scale of 0–4, measuring burden from 0 (never) to 4 (nearly always). The tool consisted of 22 questions with the maximum sum score of burden being 88 points. Scores of 0–20 indicated little or no burden; scores of 21–40 indicated mild-to-moderate burden; scores of 41–60 indicated moderate-to-severe burden; and scores of 61–88 represented severe burden.^16^ In addition, a socio-demographic information sheet, which captured caregiver demographic data as well as education, health status, social grant access, employment and family income data, was administered.

### Data analysis

Data were analysed using the Statistical Package for Social Sciences (SPSS), version 25 (IBM Corp., 2017. IBM SPSS Statistics for Windows, Armonk, New York: IBM Corp. available at: https://hadoop.apache.org.). Descriptive statistics, such as means and standard deviations, were calculated for continuous variables. Categorical variables were presented with frequency distribution. Fischer’s exact test was used to test the associations between demographic data and ZBI mean scores. All the tests were two-tailed. Statistical significance was set at *p* < 0.05.

### Ethical consideration

Ethical clearance was obtained from the University of KwaZulu-Natal’s Biomedical Research Ethics Committee (BFC386/17), further approval was granted by the relevant site authorities.

## Results

A total of 38 caregivers of CLHIV were interviewed. The demographic profiles of caregivers are reported in Table 1, with the composite burden of care reflected. The mean age of the caregivers was 33.55 (SD = 8.09) years. Seventeen of the caregivers (44.7%) reported no burden, while 14 (36.8%) of the caregivers reported mild-to-moderate burden and 7 (18.4%) caregivers felt a moderate-to-severe burden of caring for CLHIV. The majority of the study participants were females and the biological mothers (27) of the children. There were only three fathers. The rest of the caregivers were the sister (4) or grandmother (4) of the child. Most of the study participants (78.9%) were educated at a secondary school level. Most of the participants were unemployed but received some level of financial social support. Thirty-three caregivers received a child support grant and only three of the caregivers were not in receipt of any grant. Most of the participants were single (84%), while three were married and three were widowed. The majority of the participants (25) reported being in good health, while only two reported poor health. The majority of the participants (26) reported receiving support from their families more than friends and only two participants reported having no support at all.

**TABLE 1:** Demographic profile and burden of care scores.

Variables	Frequency	%
**Sex**		
Female	35	92.1
Male	3	7.9
**Relationship with child**		
Father	3	7.9
Grandmother	4	10.5
Mother	27	71.1
Sister	4	10.5
**Marital status**		
Married	3	7.9
Single	32	84.2
Widowed	3	7.9
**Health status**		
Fair	11	28.9
Good	25	65.8
Poor	2	5.3
**Employed**		
No	26	68.4
Yes	12	31.6
**Monthly income**		
< R2000.00	22	57.9
> R5000.00	7	18.4
R2000.00 – R5000.00	9	23.7
**Grant**		
No	2	5.3
Child support grant	33	86.8
Pension and other grant	3	7.9
**Support system**		
Family	26	68.4
Friends	2	5.3
None	10	26.3
**Educational level**		
Illiterate	2	5.3
Primary school level	6	15.8
Secondary school	14	36.8
Tertiary level and above	16	42.1
**Zarit Burden questionnaire composite scores**		
**Burden**		
No burden	17	44.7
Mild-to-moderate burden	14	36.8
Moderate-to-severe burden	7	18.4

Caregiver demographic data were cross-tabulated with the level of burden of care. The only significant association evidenced was between the level of burden of care and caregiver health status (*p* = 0.034). Table 2 shows the association between the demographic profile variables and the burden of care.

**TABLE 2:** Relationship of variables with severity of burden of care.

Variables	Burden						Fisher’s exact *p*
	No burden		Mild–to-moderate burden		Moderate-to-severe burden		
	*n*	%	*n*	%	*n*	%
**Sex**							0.803
Female	15	42.9	13	37.1	7	20.0	
Male	2	66.7	1	33.3	0	0.0	
**Relationship with child**							0.668
Father	2	66.7	1	33.1	0	0.0	
Grandmother	1	25.0	2	50.0	1	25.0	
Mother	11	40.7	11	40.7	5	18.5	
Sister	3	75.0	0	0.0	1	25.0	
**Marital status**							0.091
Married	1	33.3	2	66.7	0	0	
Single	16	50.0	9	28.1	7	21.9	
Widowed	0	0.0	3	100	0	0.0	
**Health status**							0.034
Fair	2	18.2	7	63.6	2	18.2	
Good	15	60.2	6	24.0	4	16.0	
Poor	0	0.0	1	50.0	1	50.0	
**Employed**							0.999
No	12	46.2	9	34.6	5	19.2	
Yes	5	41.7	5	41.7	2	16.7	
**Monthly income**							0.403
< R2000.00	9	40.9	10	45.5	3	13.6	
> R5000.00	3	42.9	3	42.9	1	14.3	
R2000.00 – R5000.00	5	55.6	1	11.1	3	33.3	
**Grant**							0.202
No	1	50.0	0	0.0	1	50.0	
Child support grant	13	39.4	14	42.4	6	18.2	
Pension and other grant	3	100	0	0.0	0	0.0	
**Support system**							0.771
Family	12	46.2	10	38.5	4	15.4	
Friends	1	50.0	0	0.0	1	50.0	
None	4	40.0	4	40.0	2	20.0	
**Educational level**							0.127
Illiterate	1	50.0	1	50.0	0	0.0	
Primary level	2	33.3	2	33.3	2	33.3	
Secondary level	10	71.4	2	14.3	2	14.3	
Tertiary level and above	4	25.0	9	56.3	3	18.8	

## Discussion

This study sought to investigate the level of caregiver burden among caregivers of CLHIV who may be experiencing disabilities. Family caregivers of CLHIV have consistently been identified as vulnerable to economic hardship, decreased mental and physical health and increased burden of care.^2,7,17^ Although some of the caregivers in this study reported having little or no burden, most caregivers reported experiencing a level of burden from mild to moderate and moderate to severe. Other sub-Saharan studies^15,18^ demonstrated lower levels and degrees of burden in larger cohort studies, but these results could possibly be attributed to the fact that most of the caregivers in those studies were not the biological parents of the CLHIV, but were trained caregivers from the community around the home.

The majority of the caregivers in this study were females, who were the biological mother, grandmother or older sister of a child living with HIV. In addition, most of the female participants were unmarried and not in relationships, with only three of the mothers married at the time of this study. These findings are consistent with the national statistics which reflect the high percentage of single mothers in SA.^11^ Only three males were caregivers to CLHIV in this study. Females were predominantly the primary caregivers, which is consistent with the results of other African studies,^7,9,10,15,18^ where women were seen to assume the primary role of caregiving. The need for men to assume greater responsibility in the caregiving of CLHIV has been emphasised in other studies,^4,19^ as it has implications for the emancipation of women and their economic empowerment.^7,19^ This concept is particularly pertinent in SA, to challenge the gender-based disparities that cultural beliefs and stereotypes impose on women and young girls in our context.

Despite the fact that most of the caregivers in our study were educated at secondary school level and beyond, still the majority were unemployed and living on financial support accessed through social grants or pensions. Furthermore, many of the caregivers lived on an income of less than R2000 a month, which is below the recommended minimum salary in SA, which is approximately R3440, based on a rate of R20 per hour for a 40-h week.^20^ Most current literature in Africa cites a lack of finance as one of the strongest predictors of caregiver burden, yet of those unemployed participants, many reported having little or no burden. Perhaps the low burden among unemployed caregivers might be attributed to the lack of competing work demands on the participants, or the additional support they indicated they received from family members. Family support has been regarded as having a positive influence on the caregivers, contributing to their ability to adapt positively to caring for children or adults who are unwell.^21^ Furthermore, social support in any form has been found to have a positive effect on the overall health status of the caregiver.^22,23^

While it was encouraging that most participants were in receipt of a childcare social grant, two caregivers did not receive a grant and, unfortunately, the reasons for this were not established. Many studies draw attention to the need for enhanced caregiver access to social grants.^7,19,24^ The procedural pathways and delayed processing times for grant applications have been found to pose barriers to caregivers.^17^ The need for greater mobilisation of social workers in communities may be a possible solution to address this challenge. Kidman and Heymann^9^ drew attention to the apparent lack of social workers, per capita, in SA. The authors believe that expanding social services for families affected by HIV is essential, so that they are linked to the necessary social protection, and families are protected from imminent social threats.^9^ Mafune et al.^19^ found that caregivers’ access to social grants was challenged, considerably straining their ability to adequately provide for the daily sustenance and healthcare needs of CLHIV. Food security and access to healthcare are the most rudimentary requirements for survival for CLHIV, and should at no time be compromised by access to social support, particularly in a resource-poor context.

The only variable that had a significant association with caregiver burden in this study was the health status of the caregiver. Because the vast majority of the participants were the mothers of CLHIV, and the fact that HIV infection in children is predominantly vertically acquired,^25^ we can assume that these mothers were probably, themselves, living with HIV. An earlier study on caregiver burden showed that HIV-related illness has the greatest connection with caregiver burden, even more than the burden associated with death in the family.^9,10^ Many other studies validate the direct association between caregiver health status and caregiver burden.^6,17,26^ Caregiver health status has significant implications for employment opportunities and earning potential, as well as the functional ability to carry out the caregiving tasks of daily living, understandably affecting caregiver burden.^6,7^

Increased caregiver burden has direct negative effects on the nature and quality of care offered and decreases the likelihood of the responsive caregiving.^27,28^ Responsive caregiving is associated with significant improvements in children’s outcomes across their domains of functioning.^29^ In view of the factors that influence the care burden on caregivers in a South African context, and the potential impacts on CLHIV in resource-poor settings, the need to care for the carer to improve the care offered to CLHIV is essential. Previous caregiver studies assert the need for caregiver burden screening by healthcare professionals to integrate interventions aimed at addressing these determinants in the care offered to CLHIV.^9,10,19^

Family-centred models where concerns (health or otherwise) pertaining to both the child and the parent are central to the care approach demonstrate improved outcomes for the well-being of the entire family unit.^30,31^ Routine evaluation and monitoring of caregiver burden are fitting in this framework.

## Conclusion

The majority of the caregivers in this study experienced a degree of burden between mild to moderate and moderate to severe. This was most significantly associated with the health status of the caregiver. Because caregiver well-being may affect the care offered to CLHIV, further research should longitudinally investigate caregiver burden and its implications in caregivers of CLHIV in resource-poor settings to develop appropriate interventions to reduce the load of caregiving.

## Limitations

There are several limitations to this study. The results are based on self-reporting by the caregivers, which may be biased. The cross-sectional design and small non-probability sample size, limited to a single-study site, suggest that the results cannot be generalised to other populations. The correlation between variables does not prove any causality. Furthermore, the sample was predominantly female; therefore, generalisations to male caregivers should be made circumspectly.
