# Erratum: Burden of caring for children living with human immunodeficiency virus in a semi-rural South African community

**DOI:** 10.4102/safp.v63i1.5379

**Published:** 2021-11-16

**Authors:** Stacy Maddocks, Verusia Chetty

**Affiliations:** 1 Discipline of Physiotherapy, School of Health Sciences, University of KwaZulu-Natal, Durban, South Africa

In the version of this article initially published, Maddocks DK, Chetty V. Burden of caring for children living with human immunodeficiency virus in a semi-rural South African community. S Afr Fam Pract. 2020;62(1), a5110. https://doi.org/10.4102/safp.v62i1.5110, the first author’s initials were given incorrectly in the ‘How to cite this article’ section. The correct initials should be S instead of DK.

This correction does not alter the study’s findings of significance or overall interpretation of the study’s results. The publisher apologises for any inconvenience caused.

